# Pituitary-adrenal axis dysfunction induced by tislelizumab immunotherapy for non-small cell lung cancer: a case series and literature review

**DOI:** 10.1186/s12890-024-03140-4

**Published:** 2024-07-08

**Authors:** Jiayi Wang, Huanrong Lan, Xiaowei Mao, Yefeng Chen

**Affiliations:** 1 School of Medicine, Shaoxing University, Shaoxing, 312000 Zhejiang P. R. China; 2 Department of Surgical Oncology, Hangzhou Cancer Hospital, Hangzhou, 310002 Zhejiang China; 3 Department of Pulmonary and Critical Care Medicine, Regional Medical Center for National Institute of Respiratory Diseases, Sir Run Run Shaw Hospital, School of Medicine, Zhejiang University, Hangzhou, 310016 China; 4 Department of Pulmonary Medicine, Shaoxing People’s Hospital, Shaoxing, 312000 China

**Keywords:** Anti-PD-1 monoclonal antibody, Pituitary inflammation, Adrenal dysfunction, Pulmonary malignancy, Immune checkpoint inhibitors

## Abstract

**Background:**

Adverse events of secondary adrenal insufficiency caused by anti-PD-1 immune agents are relatively rare in clinical practice, so in this article, we retrospectively analyzed three patients who suffered secondary adrenal cortex dysfunction caused by tislelizumab immunotherapy for Non-Small Cell Lung Cancer (NSCLC)and reviewed the literature. This rare immune-related adverse event was investigated by summarizing the clinical features of the patients.

**Case presentation:**

We reported three NSCLC patients who suffered secondary adrenal cortex dysfunction induced by tislelizumab immunotherapy at our hospital from July 2021 to October 2023. We analyzed and summarized the clinical characteristic, laboratory examination, pathological staging, etc. We also reviewed related literature of pituitary inflammation and adrenal cortex dysfunction caused by immunotherapy.

**Results:**

The median age of the three patients was 56 years. All the patients had a history of smoking. After receiving tislelizumab treatment (median cycle: 7), laboratory examination showed a decrease in morning cortisol and adrenocorticotropic hormone (ACTH), both were diagnosed with secondary adrenal insufficiency. Only one patient had symptoms of fatigue, vomiting, and weight loss. One of these patients also had simultaneous subclinical hypothyroidism. All three patients discontinued immunotherapy and received replacement therapy with glucocorticoids. At the last follow-up, none of the three patients restarted immunotherapy, because cortisol did not return to normal. This is similar to that of previous reports.

**Conclusion:**

Based on previous reports and our three cases, when laboratory tests of NSCLC patients receiving immunotherapy showed a decrease in morning cortisol and ACTH levels, especially when clinical symptoms were obvious, the possibility of immunotherapy-related pituitary inflammation causing secondary adrenal cortex dysfunction should be considered. Prompt monitoring and hormone replacement therapy should be provided to prevent adrenal crises.

## Background

According to the National Cancer Center of China, lung cancer had the highest morbidity and mortality in 2020 [1, 2], with NSCLC accounting for approximately three-quarters of the cases. The treatment options for NSCLC include surgical resection, chemotherapy, immunotherapy, target therapy, etc. In the past few years, immunotherapy, either alone or combined with other treatments, shown improved survival rate of NSCLC patients [3]. The most commonly drugs applied in clinical are programmed death protein 1 (PD-1), programmed death ligand 1 (PD-L1) inhibitors and anti-cytotoxic T lymphocyte-associated antigen 4 (CTLA-4) inhibitors. PD-1 and CTLA-4 are receptors expressed on the membrane of cytotoxic T lymphocytes and exert antitumor effects by blocking the escape of tumor cells caused by the binding of tumor surface markers to these receptors [4].Tislelizumab is a humanized IgG4 anti–PD-1 monoclonal antibody specifically designed to minimize binding to FcγR on macrophages. The RATIONALE-307 studied the safety and efficacy of chemotherapy puls tislelizumab as first-line therapy in advanced NSCLC patients. It found chemotherapy puls tislelizumab had longer median progression-free survival (PFS) over 7.6 months.Meanwhile, the AE was similar in experiment group or control group.Based on this study, the National Medical Products Administration (NMPA) approved tislelizumab combined with chemotherapy as the first-line treatment of advanced squamous NSCLC [5]. The RATIONALE-303 studied compred the safety and efficacy of tislelizumab or docetaxel in previously treated advanced NSCLC patients.The results showed the tislelizumab group had more longer median overall survival (OS) over 16 months.So the NMPA approved tislelizumab as second- or third-line treatment of patients with locally advanced or metastatic NSCLC [6]. 

While immune checkpoint inhibitors have shown excellent anticancer efficacy, immune-related adverse events (irAEs) should not be ignored. These adverse events can affect almost all systems of the human body. It may involve the lungs, heart, kidneys, skin, pituitary gland, and other organs [7, 8]. Since immune checkpoint inhibitors regulate the immune system to fight against tumors, immune-related side effects are unavoidable. However, during the treatment, it is crucial to identify these irAEs early and respond quickly, as it can be life-threatening [9]. Several studies have suggested [10, 11] that endocrine-related irAEs are the most common irAE. However, most stuies focused on thyroid dysfunction, type 1 diabetes, few irAE of pituitary-adrenal axis was reported. The pituitary-adrenal axis regulates the release of glucocorticoids, sex hormones, and other adrenocortical hormones by releasing ACTH. Therefore, pituitary inflammation can cause various hormone abnormalities. Adrenal dysfunction secondary to pituitary inflammation can be life-threatening if it cannot be detected timely. These irAEs should be detected early and respond quickly in clinical practice. In this study, we report three cases of pituitary inflammation caused by tisilizumab treatment and secondary adrenocortical insufficiency. Additionally, we reviewed the previous literature to summarize the clinical characteristics of this irAE.

## Case presentation

### Case 1

Case 1, a 51-year-old male with a long history of heavy smoking, was diagnosed with 8th TNM stage IVA (cT4N3M1a) right lung squamous cell carcinoma (Table 1). The patient had enlarged mediastinal and hilar lymph nodes. After receiving four cycles of first-line chemotherapy (albumin-bound paclitaxel plus carboplatin) combined with tislelizumab immunotherapy (Table 2),in order to continue his fifth cycle of immunotherapy, the patient was admitted to the hospital for evaluation of his condition. The patient did not experience significant weight loss, headache, fatigue, or memory decline.

**Table 1:** Patient characteristics

Case	Age	Diagnostic	8th TNM Stage	Tobacco habit
1	51	squamous cell carcinoma in right lung	IVA(cT4N3M1a)	30 years,1packs /day
2	74	squamous cell carcinoma in left lung	IVA(cT4N0M1b)	40 years, 2packs /day
3	56	squamous cell carcinoma in right lung	IIIA(pT2aN2M0)	20 years, 1packs /day

**Table 2:** Clinical, biochemical, and image alterations of patients with secondary adrenocortical insufficiency

Case	Treatment received	Onset time	Onset Laboratory indicators	Onset symptoms	Pituitary MRI	Treatment
1	albumin-bound paclitaxel plus carboplatin and Tislelizumab^1^	Cycle 4	Cortisol and ACTH decreased synchronously, Thyroid-stimulating hormone was increased, FSH, prolactin, and serum growth hormone were increased, and insulin-like growth factor-1 was decreased	unspecific	negative for hypophysitis	Prednisone acetate 5 mg bid replacement therapy and Euthyrox 50ug qm supplementation
2	Tislelizumab^2^	Cycle 9	Cortisol and ACTH decreased synchronously	unspecific	not performed	Prednisone acetate 5 mg AM,2.5 mg PM replacement therapy
3	albumin-bound paclitaxel plus carboplatin and Tislelizumab^3^	Cycle 8	Cortisol and ACTH decreased synchronously	fatigue, vomiting, and weight loss	not performed	Prednisone acetate 5 mg AM replacement therapy

^1^albumin-bound paclitaxel 200 mg (day 1、day 8) plus carboplatin 550 mg and Tislelizumab 200 mg every 21 days. ^2^Tislelizumab 200 mg every 21 days. ^3^albumin-bound paclitaxel 200 mg (day 1、day8) plus carboplatin 500 mg and Tislelizumab 200 mg every 21 days for the first 4 cycles, maintaining with single drug Tislelizumab 200 mg every 21 days.

**Fig. 1:**
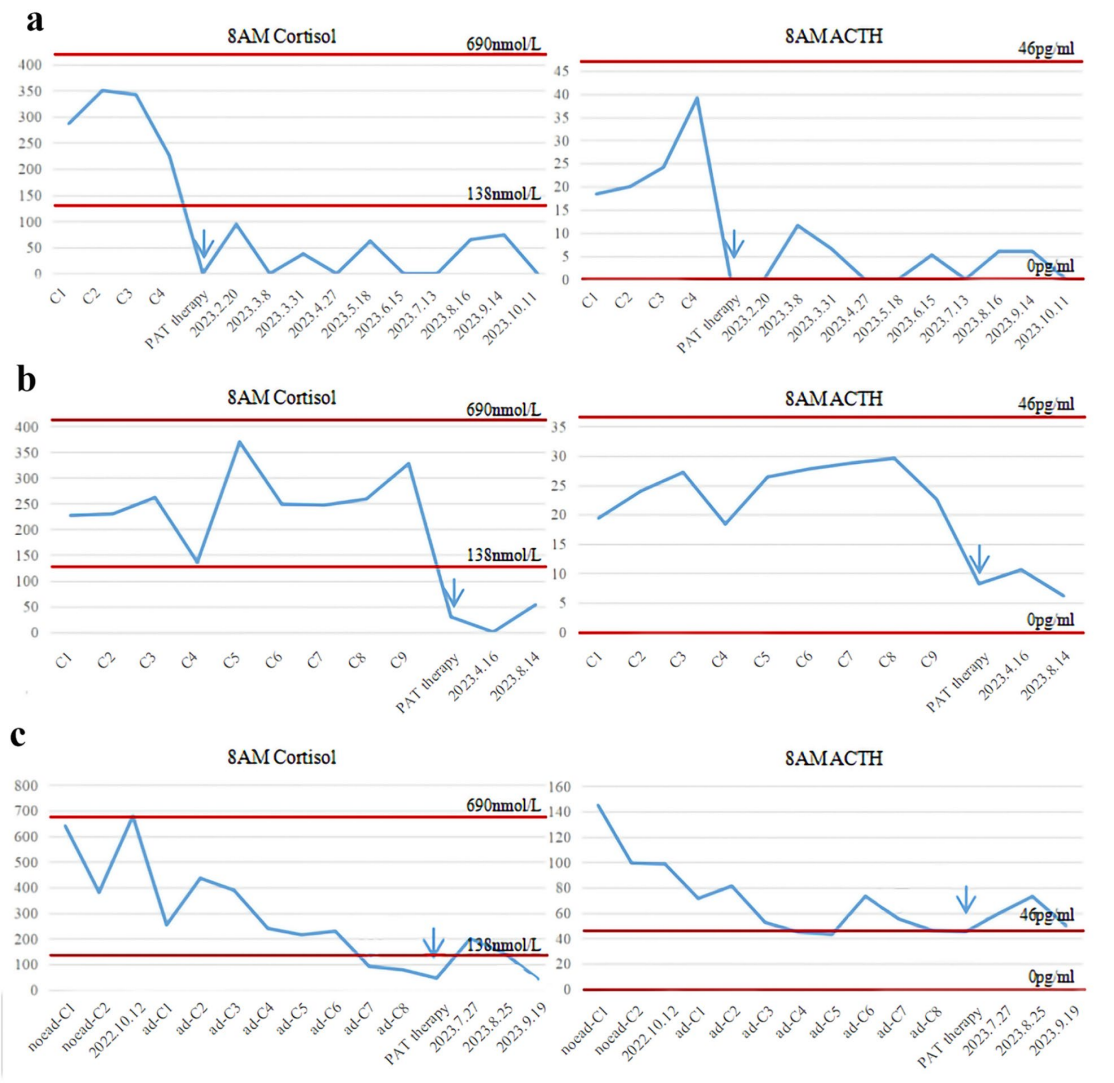
**a** It is cortisol and ACTH during Case1 immunotherapy, and the time indicated by the arrows indicates that the patient discontinued immunotherapy and started prednisone acetate replacement, followed by the retention of the time for each follow-up visit. **b** It is cortisol and ACTH during Case2 immunotherapy, and the time indicated by the arrows indicates that the patient discontinued immunotherapy and started prednisone acetate replacement, followed by the retention of the time for each follow-up visit. **c** It is cortisol and ACTH during Case3 immunotherapy, the patient first received two cycles preoperative neoadjuvant immunotherapy, October 12, 2022 is the laboratory result of the patient before surgery, and the time indicated by the arrow indicates that the patient stopped immunotherapy and started prednisone acetate replacement, and then retained the time for each follow-up visit. *All data were collected 1–2 days prior to the planned immunotherapy cycle. Abbreviations: PAT: Prednisone acetate; C1:first cycle of treatment; noead: Neoadjuvant immunotherapy; ad: Adjuvant immunotherapy

Physical examination revealed no obvious abnormalities. Weight was not obviously decreased.The auxiliary examinations suggested that his 8 am cortisol and 8 am ACTH levels decreased before admission (Fig. 1a), his thyroid-stimulating hormone levels increased, and his liver and kidney function, electrolytes and fasting glucose levels were normal. The chest CT enhancement suggested that the enlarged mediastinal lymph nodes improved after the previous examination.Clinical pituitary function stimulation tests were not performed.

Further hormone testing, including routine reproductive hormone tests, suggested that follicle-stimulating hormone, prolactin, and serum growth hormone levels were elevated, while insulin-like growth factor-1 was decreased. Pituitary magnetic resonance imaging showed no abnormalities. After multidisciplinary team discussion involving an endocrinologist, the patient was considered to have “secondary adrenal insufficiency”, and immunotherapy was deferred. The patient continued with chemotherapy (albumin-bound paclitaxel plus carboplatin) and received acetate prednisone replacement therapy and Euthyrox supplementation. After more than 2 months of Euthyrox supplementation, his thyroid indices returned to normal. Hormone replacement therapy had been ongoing for more than 8 months, and cortisol levels measured in the morning on a monthly basis had consistently been lower than normal. As of the last follow-up, the patient was still below normal levels of cortisol, so immunotherapy had not been restarted.

## Case 2

Case 2, a 74-year-old male, had a history of hypertension and a long history of smoking (Table 1). He was diagnosed with left lung squamous cell carcinoma (8th edition TNM stage IVA, cT4N0M1b), accompanied by rib metastasis. He received one cycle of treatment with albumin-bound paclitaxel plus carboplatin and tislelizumab, which resulted in a rash all over his body. He then underwent five cycles of gemcitabine plus carboplatin chemotherapy, followed by five cycles of gemcitabine monotherapy. After assessing the tumor condition, a chest enhanced CT indicated tumor progression. He then received nine cycles of third-line tislelizumab monotherapy (Table 2). Due to progression of the tumor to bone destruction, he received one dose of tislelizumab plus denosumab. He was admitted to the hospital in order to continue his immunotherapy after 21 days. The patient had not experienced significant weight loss, headache, fatigue, memory decline, or other symptoms.

Physical examination revealed no obvious abnormalities. Weight was not obviously decreased. The auxiliary examinations suggested that his 8 am cortisol and 8 am ACTH levels decreased after admission (Fig. 1b). Thyroid function, liver and kidney function, electrolytes and fasting glucose were normal. The chest CT enhancement suggested that the lung lesions were similar to those observed during the previous examination.Clinical pituitary function stimulation tests were not performed.

After discussion with the multidisciplinary team, which included an endocrinologist, the patient was diagnosed with “adrenal insufficiency,” and the patient received acetate prednisone replacement therapy. Immune checkpoint inhibitor-induced adverse reactions were considered, and immune therapy was deferred. Hormone replacement therapy had been ongoing for more than 7 months, and the morning fasting cortisol level remained significantly below normal. At the last follow-up, immune therapy had not resumed.

## Case 3

Case 3 was a 56-year-old male with a history of diabetes and a long history of heavy smoking (Table 1). He was diagnosed with squamous cell carcinoma of the right lung (8th edition TNM stage IIIA, cT4N1M0) along with enlarged right hilar lymph nodes. After treatment with albumin-bound paclitaxel, carboplatin, and tislelizumab, the lung lesions shrunk, and a local cavity formed, as observed via a three-dimensional CT scan. The patient underwent right middle lobectomy combined with mediastinal lymph node resection. Postoperatively, restaging revealed a malignant tumor in the right middle lobe (pT2aN2M0, stage IIIA). The patient received chemotherapy (albumin-bound paclitaxel plus carboplatin) in combination with tislelizumab immunotherapy for four cycles, followed by four cycles of immunotherapy as a monotherapy (Table 2). Before the ninth cycle of treatment, the patient was admitted to the hospital for evaluation and was found to have symptoms such as fatigue, vomiting, and weight loss.

Physical examination revealed that the patient had multiple red rashes with itching of the skin. His weight decreased by 2.5 kg in one month. The auxiliary examinations suggested that his 8 am cortisol and 8 am ACTH levels decreased after admission (Fig. 1c). Thyroid function, liver and kidney function, electrolytes and fasting glucose were normal.Clinical pituitary function stimulation tests were not performed.

After a multidisciplinary team discussion involving an endocrinologist, the patient was diagnosed with “adrenal insufficiency”, and replacement therapy was started with hydrocortisone acetate. After 2 months of replacement therapy, his cortisol levels normalized, and his ACTH levels increased compared to those at the previous examinations. The patient was readmitted on August 28, 2023, and received one cycle of maintenance treatment with tislelizumab monotherapy. His cortisol levels decreased again to 43 nmol/L, and immunotherapy was discontinued, with steroid replacement reinitiated. As of the last follow-up, immunotherapy had not been restarted.

## Discussion

With the widespread using of immune checkpoint inhibitors in NSCLC clinical practice, more patients experience varying degrees of irAEs. Among all irAEs, pituitary-adrenal axis dysfunction was not common but was life-threatening. In our center, three patients exhibited adrenal insufficiency after tislelizumab immunotherapy. The morning cortisol and ACTH levels were decreased in all three patients (Fig. 1). The adenohypophysis mainly affects three glandular axes, and we mainly discuss the adrenal axis in this manuscript. In the clinical practise, the cortisol and ACTH in three patients showed a downward trend, and one patient also showed changes in the thyroid, so it was considered that the tislelizumab may result hypopituitarism,. We consider that the early detecting hormonal changes, the influence of immunotherapy was limited to adrenal axis. Besides, weather the adrenal axis was more susceptible to attacks by immunotherapy, this need more clincal studies to answer. Although the first two patients did not show obvious clinical manifestations, cortisol and ACTH decreased significantly and rapidly. After the MDT including the endocrinology department, we decided to temporarily stop the immunotherapy.

Several studies have suggested [10, 12, 13] that PD-1 and PD-L1 immune checkpoint inhibitors can cause adrenal insufficiency, which can be categorized as primary or secondary pituitary inflammation. Clinical symptoms of adrenal insufficiency include fatigue, nausea, and weakness. Primary adrenal insufficiency is often the direct result of immune therapy damaging the adrenal glands, result to hormonal abnormalities. Laboratory examination showed decreased morning cortisol and increased ACTH levels. On the other hand, secondary adrenal insufficiency is commonly associated with pituitary inflammation, which affects adrenal hormone secretion through the pituitary-adrenal axis and manifests as a synchronous decrease in morning cortisol and ACTH levels.The consequences of pituitary gland damage caused by immunotherapy, adrenal hormone and thyroid hormone disorders are more serious and the incidence is not uncommon, so we routinely test cortisol and ACTH in non-small cell lung cancer patients before the chemotherapy and immunotherapy in each treatment cycle.

In the three patients, the cortisol and ACTH levels decreased synchronously, and one patient exhibited thyroid dysfunction and elevated sex hormone levels meantime. Although pituitary MRI showed no abnormalities, pituitary inflammation cannot be excluded. It has been reported [13–15] that early or mild pituitary inflammation cases may be normal on pituitary MRI. Retrospective studies [16] have shown that most patients receiving CTLA-4 therapy may have abnormal pituitary MRI results, while only a small number of patients receiving PD-1 and PD-L1 therapy may experience transient pituitary enlargement. Therefore, based on the laboratory results and auxiliary examinations, immune therapy-induced pituitary inflammation was considered the cause of secondary adrenal cortex dysfunction in these three patients.

Immune checkpoint inhibitors mainly block ligands on tumor cells and thereby may enhance the ability of T cells to kill tumor cells. Tislelizumab, a humanized IgG4 anti-PD-1 monoclonal antibody,. PD-1 prevents autoimmune responses by promoting the apoptosis of antigen-specific T cells in lymph nodes and reducing the apoptosis of regulatory T cells [17]. However, PD-1 expressed on the tumor cells surface reduces the aggressiveness of T cells through this pathway, enabling immune escape. Therefore, the application of anti-PD-1 antibodies in immune therapy inevitably enhances the aggressiveness of T cells, leading to irAEs [10].The RATIONALE-303 study showed the incidence of all grades of treatment-related about adverse events (TRAEs) treated with tislelizumab was 18.9%, and hypothyroidism (7.9%), pneumonia and immune-mediated lung disease (4.5%) were the most TRAEs [6].

During immunotherapy, irAEs in the endocrine system are common [12]. However, pituitary inflammation is relatively rare, especially in patients treated with PD-1 or PD-L1 alone [18, 19]. In RATIONALE-303/307, not detail of pituitary inflammation reported. It is more common in patients receiving anti-CTLA-4 immunotherapy, particularly in those receiving ipilimumab [20], and may be associated with high CTLA-4 expression in the pituitary [21]. The secretion of glucocorticoids and mineralocorticoids by the adrenal cortex is vital for the body’s metabolism, immune function, and water-electrolyte balance. Early detection of adrenal crisis is clinically important if adrenal cortical insufficiency occurs.

Among the three patients, the first patient received first-line chemotherapy combined with immunotherapy, the second patient received third-line monotherapy immunotherapy, and the third patient received perioperative immunotherapy as neoadjuvant and postoperative adjuvant treatment. The median time from the start of immunotherapy was 7 cycles, and the median number of immunotherapy cycles was seven. Previous reports suggest that pituitary inflammation caused by PD-1/PD-L1 immune checkpoint inhibitors typically occurs after seven cycles of treatment [19], which is consistent with our reports. Laboratory tests [22, 23] indicated decreased morning cortisol and ACTH levels in all three patients, but only the third patient exhibited clinical symptoms of fatigue, anorexia, and vomiting. Case 3 suffered vomiting and weight loss, which were more substantial than in the other two cases. However, in Case 3, serum cortisol decreased subclinical levels.We considered to be related to tumor stage and individual differences.

Clinical symptoms of anti-PD-1-related pituitary inflammation often manifest as nonspecific symptoms such as fatigue, nausea, and blurred vision [24, 25]. These irAEs tend to involve the adrenal cortex axis. Regular monitoring of adrenal cortex-related hormones is necessary for patients with atypical clinical symptoms. If cortisol and ACTH levels decrease, along with thyroid hormone or sex hormone levels, the possibility of pituitary inflammation should be considered. Immune-related pituitary inflammation can have varying effects on the damage and recovery of the adrenal axis, thyroid axis, and gonadal axis. Patients with hormone deficiency caused by adrenal axis damage often have difficulty recovering [26–28]. Therefore, regular monitoring of hormone levels is crucial, and if necessary, pituitary MRI should be performed to evaluate pituitary lesions. Immunotherapy was discontinued in all three patients, and hormone replacement therapy was initiated.

The clinical practice guidelines of the American Society of Clinical Oncology for irAEs caused by immune checkpoint inhibitors suggest that patients with grade 1–2 irAEs who have mild or recoverable symptoms can receive hormone replacement therapy without stopping immune treatment. However, for Grade 3 or higher irAEs, high-dose steroid pulse therapy is required when the patient develops severe symptoms, and immunotherapy should be temporarily discontinued [13]. However, Elia Seguí proposed that grade 1 irAEs do not require stopping immune treatment, grade 2 irAEs necessitate treatment cessation and close monitoring, and grade 3 irAEs require hospitalization and treatment with high-dose corticosteroids in addition to immune treatment cessation [4]. In addition, high-dose corticosteroid replacement therapy requires gradual tapering and remains long-term in most patients after symptoms have resolved and laboratory markers have stabilized [29].

Even for the three patients with either no symptoms or mild symptoms, their morning cortisol and ACTH levels were significantly lower, indicating adrenal insufficiency. To prevent adrenal crisis, prednisolone acetate was administered in a timely manner, and immune treatment was postponed. However, the recovery of cortisol and ACTH levels was not significant in any of the three patients, and at the last follow-up, the cortisol levels were not normal. Therefore, immune treatment was not resumed. Despite the occurrence of many irAEs, several studies have shown that NSCLC patients who experience adverse reactions have higher response rates, disease control rates, progression-free survival, and overall survival than do those who do not experience adverse reactions [13, 30–32].Update the follow-up data, no tumor recurrence or metastasis was found in these three patients.

## Conclusion

Combined with the case series and literature review, the irAE of pituitary-adrenal axis dysfunction was relatively rare in the treatment of anti-PD-1, and the symptoms were usually non-specific manifestations, e.g. nausea, fatigue, and vomiting. Considering that the asymptomatic hypophysitis may be life-threatening, when laboratory tests showed decreased morning cortisol and ACTH levels, regardless of the presence of clinical symptoms, the possibility of pituitary inflammation should be considered. We recommend that regular laboratory test results should be conducted during every cycle from the initiation of immune treatment to early detect of adrenal cortex dysfunction. Hormone replacement therapy may be the best treatment for pituitary-adrenal axis dysfunction.

## Data Availability

The datasets used and/or analysed during the current study are available from the corresponding author on reasonable request.

## Abbreviations

NSCLC: non-small cell lung cancer
ACTH: adrenocorticotropic hormone
PD-1: programmed death protein 1
PD-L1: programmed death ligand 1
CTLA-4: anti-cytotoxic T lymphocyte-associated antigen 4
PFS: progression-free survival
NMPA: National Medical Products Administration
OS: overall survival
irAEs: immune-related adverse events
TRAEs: treatment-related about adverse events
